# Supplementary material for: The causal relationship between the human gut microbiota and pyogenic arthritis: a Mendelian randomization study
Source: Front Cell Infect Microbiol. 2024 Nov 26;14:1452480. doi: 10.3389/fcimb.2024.1452480 (PMC11629706; doi:10.3389/fcimb.2024.1452480)
Supplement: Supplementary file 1 [file DataSheet1.zip › mendelian test/ebi-a-GCST90027480.csv_forest.pdf]

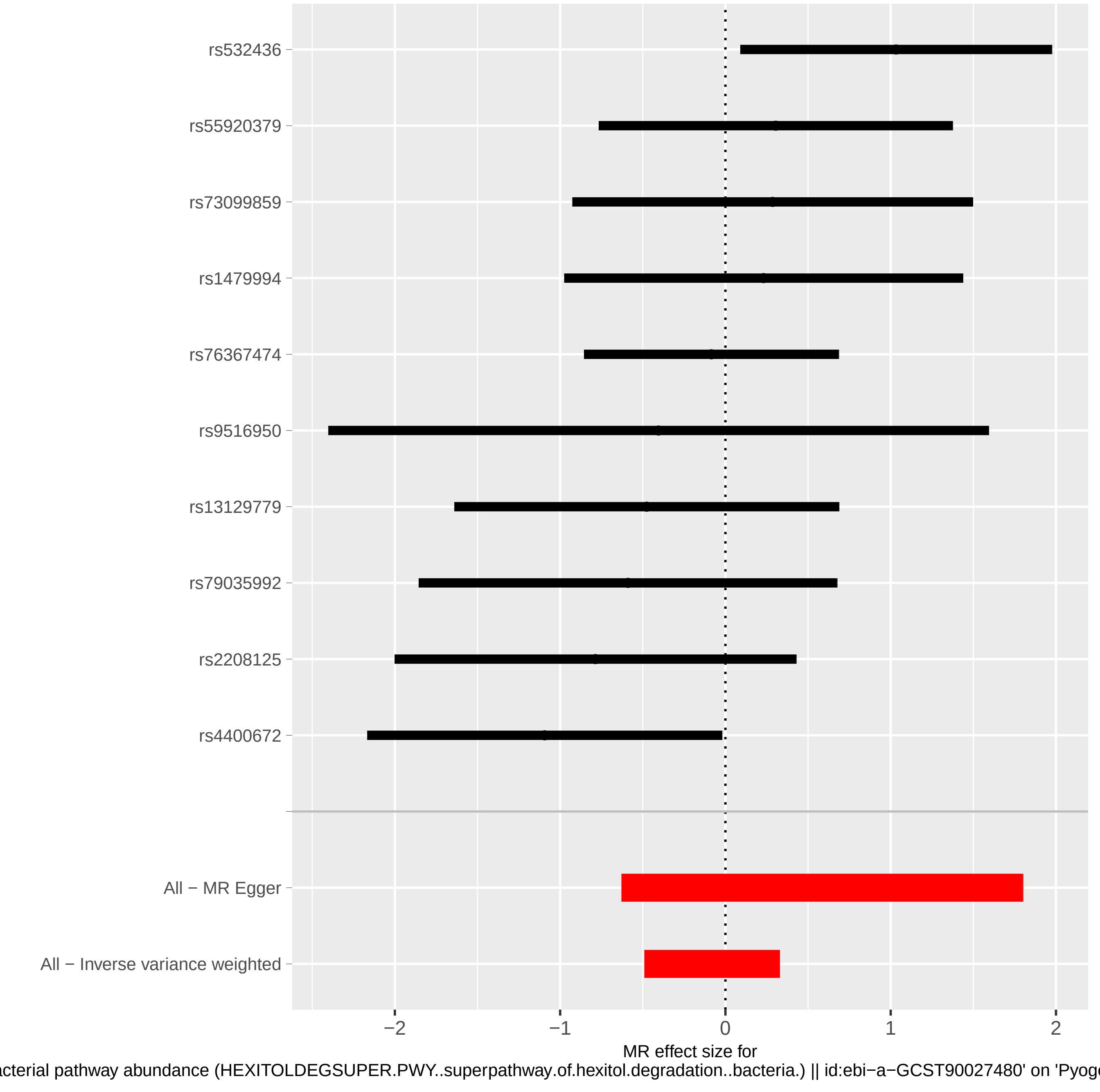
rs532436
rs55920379
rs73099859
rs1479994
rs76367474
rs9516950
rs13129779
rs79035992
rs2208125
rs4400672
All − MR Egger
All − Inverse variance weighted
−2
−1
0
1
2
MR effect size for
acterial pathway abundance (HEXITOLDEGSUPER.PWY..superpathway.of.hexitol.degradation..bacteria.) || id:ebi−a−GCST90027480' on 'Pyog
